# Immunohistochemical Detection of the Presence of Vitamin D Receptor in Childhood Solid Tumors

**DOI:** 10.3390/cancers14143295

**Published:** 2022-07-06

**Authors:** Orsolya Juhász, Noémi Jákob, Hajnalka Rajnai, Marcell Imrei, Miklós Garami

**Affiliations:** 1 2nd Department of Pediatrics, Semmelweis University, 1094 Budapest, Hungary; 2 1st Department of Pathology and Experimental Cancer Research, Semmelweis University, 1085 Budapest, Hungary; jakob.noemi@med.semmelweis-univ.hu (N.J.); rajnai.hajnalka@med.semmelweis-univ.hu (H.R.); 3 Centre for Translational Medicine, Semmelweis University, 1085 Budapest, Hungary; marcell.imrei@gmail.com; 4 Heim Pál National Pediatric Institute, 1089 Budapest, Hungary

**Keywords:** vitamin D receptor, childhood cancers, tissue microarray, survival

## Abstract

**Simple Summary:**

Childhood cancer cannot generally be prevented or identified through screening. The disease is often diagnosed at advanced stages, and this obviously affects patient outcomes negatively. There is an urgent need for the identification of prognostic and therapeutic biomarkers. In this study, lower vitamin D receptor (VDR) expression was associated with a worse prognosis in various childhood cancer patients (significant correlation, *p* = 0.0061). The identification of VDR status in various childhood cancers has highlighted potential patient populations that could benefit from the newly identified biomarker. Knowledge of the VDR status in childhood solid tumors provides clinicians with a valuable biomarker and based on this, additional therapy (vitamin D and analogs) to use alongside conventional first-line oncotherapy.

**Abstract:**

Background: Our previous work has shown a correlation between lower vitamin D levels in children with cancer and adverse prognosis. It suggests that supplying vitamin D is reasonable. VDR expression in childhood solid tumors has been linked to tumor characteristics and patient survival in only a few studies. Methods: For this study, 177 children with solid tumors were selected whose biopsies and tumor tissue formalin-fixed, paraffin-embedded tissue blocks were available for immunohistochemical analysis at Semmelweis University, Budapest (Hungary). Results: We found that non-significant VDR expression was associated with a significantly less favorable prognosis (*p* = 0.0061) in the examined childhood solid tumors. There was a clinically significant association; non-significant VDR expression had more than 14-fold odds of an unfavorable prognosis (OR = 14.74). The rate of VDR expression differed significantly between tumor types (*p* < 0.0001). Conclusion: In conclusion, VDR expression measured by IHC staining is inversely associated with aggressive characteristics in different childhood cancers. The downregulation of VDR expression in more aggressive childhood cancers suggests that functional vitamin D activity may slow or block cancer progression.

## 1. Introduction

According to a multinational assessment, the incidence of most forms of childhood and adolescent cancer has increased significantly in the majority of regions over the last 40 years [1]. Despite outstanding mortality rates, cancer is the second major cause of death from disease in children beyond infancy, after accidents, in high-income nations, responsible for more than 5000 potentially avoidable deaths annually in people below 15 years of age [2]. Over the previous 50 years, childhood cancer survival has improved significantly in high-income countries worldwide [3], with estimated 5-year survival approaching 80% in several European countries [4]. During this time in Western Europe, mortality from all childhood malignancies decreased by nearly 60% while the declining trends began later and were significantly smaller (by around 30%) in Eastern European countries [5].

The literature on the connection between certain adult malignancies and vitamin D insufficiency and vitamin D signaling pathways [6,7] has grown significantly in recent decades, for example, in breast cancer [8], pancreatic cancer [9], lung cancer [10] and melanoma [10,11]. The primary active form of vitamin D, 1,25(OH)2D3, is a steroid hormone that binds to VDR in the bone tissue, kidneys and intestinal tract to increase serum calcium levels. Various cell types are able to convert it to the active 1,25(OH)2D3 form. In a variety of tumor cell types, this active form has been demonstrated to have antiproliferative and anti-invasive abilities, as well as the ability to drive apoptosis [8].

The vitamin D receptor (VDR) belongs to the transcription factor superfamily of nuclear receptors; thus, vitamin D finds a direct approach to regulate genes through 1,25(OH)2D3. VDR has been reported in different tissues and types of epithelial and mesenchymal cells [12]. VDR localization is predominantly nuclear, although VDR has been found in the cellular membrane and cytoplasm according to studies using radiolabeled 1,25(OH)2D3 [13].

In a well-known signaling pathway, VDR binds 1,25(OH)2D3 in the cell, and the resulting complex connects with the retinoid X receptor to form a 1,25(OH)2D3*VDR*retinoid X receptor heterodimer. Vitamin D-responsive elements in the promoter regions of target genes can be bound by this complex. This interaction results in a number of downstream effects, including the stimulation of differentiation and inhibition of proliferation, angiogenesis, invasiveness and metastatic potential [14].

There is a growing literature on tumor research on potentially effective complementary therapies to oncotherapy, such as the role of vitamin D through its antitumor gene-modifying effect on VDR [15,16]. One of the most widely used methods to study the presence and pattern of VDR protein expression in certain tumor tissues is immunohistochemistry [8,17,18]. However, the VDR pattern of different tumor types is a less studied area in the case of childhood malignancies [19].

The current literature and the shortcomings of pediatric oncology aspects have aroused our interest in examining VDR in pediatric malignancies. This study is the first to investigate the possible presence of VDR in pediatric tumor specimens, group entities based on the findings and to examine the role of these findings as a potential prognostic factor.

## 2. Materials and Methods

### 2.1. Patient and Sample Selection

For this study, 177 children with solid tumors were selected whose biopsies and tumor tissue formalin-fixed, paraffin-embedded (FFPE) tissue blocks were available for immunohistochemical analysis. The patients were previously treated at the 2nd Department of Pediatrics, Semmelweis University. The samples were stored, prepared and analyzed at the 1st Department of Pathology, Semmelweis University. In our study, we examined the presence of VDR in 177 childhood tumor samples and classified each tumor entity based on their VDR pattern. The analysis included 37 ependymoma, 10 Ewing’s sarcoma, 6 ganglioneuroma, 2 hepatoblastoma, 68 medulloblastoma, 27 neuroblastoma, 5 papillary thyroid carcinoma, 12 primitive neuro-ectodermal tumor (PNET), 1 rhabdoid renal tumor, 2 teratoma and 7 Wilms’ tumor samples. Diagnoses were based on histopathologic, immunophenotypic and molecular analyses according to the current World Health Organization (WHO) classification. To investigate the association between the presence of VDR in the tumor tissue and outcome, tumor samples and the survival datasets of 110 children were available. Among these 110 children, 62 (56%) were boys, and 48 (44%) were girls. The median age was 3.5 years (mean: 5.6 years) (Table S1).

### 2.2. Tissue Microarrays

After selecting representative tumor areas on the hematoxylin-eosin-stained slides, tissue microarrays (TMA) were created with a computer-driven semi-automated instrument (TMA Master, 3D HISTECH Ltd., Budapest, Hungary). Duplicate or triplicate cores of 2 mm diameter were arrayed from the tumor samples into the recipient blocks. In the case of smaller tumor samples, whole slides were used.

### 2.3. Immunohistochemical Analysis

The TMA blocks were cut into 3 µm sections and then applied to silane-coated adhesive microscope slides. Immunohistochemical staining was performed on the Dako Agilent Autostainer System (Agilent, Santa Clara, CA, USA). Antigen retrieval was performed using Target Retrieval Solution, Low pH. TMA sections were labeled with VDR antibody (Santa Cruz, mouse monoclonal antibody, 1:600 dilution). External controls (cerebral cortex, cerebellum, lymph node, liver, kidney, uterus and skin) were used as positive and negative controls. TMA slides were scanned using a Panoramic scan instrument (3D HISTECH) equipped with a 20× Carl Zeiss objective (NA = 0.83; Carl Zeiss MicroImaging Inc., Jena, Germany).

The relative proportion of positive cells was determined on the scanned slides at 400× magnification by counting 200 cells in three different areas on each TMA core by two independent pathologists, evaluating a minimum of a 0.1 mm^2^ area. According to the expression level of the VDR protein, the following scores were assigned: negative < 10% (1—low), 11–50% (2—intermediate) and 51–100% (3—high).

All tissue samples were handled in a coded fashion, according to the Dutch and Hungarian National Ethical guidelines (National Ethical Review Board approval: TUKEB no. 7/2006).

### 2.4. Statistical Analysis

Fisher’s exact test was conducted to analyze the relations between groups. The result was considered significant if the *p*-value was less than 0.05. For comparison of two groups, the odds ratio was calculated with Haldane–Anscombe correction. The proportion of significant VDR expression was calculated for the different diagnoses. Confidence intervals of 95% were assigned to the proportions, for which Wilson score intervals were calculated. All analyses were carried out by R version 4.1.0.

## 3. Results

### 3.1. Measurement of VDR Expression

**Figure 1:**
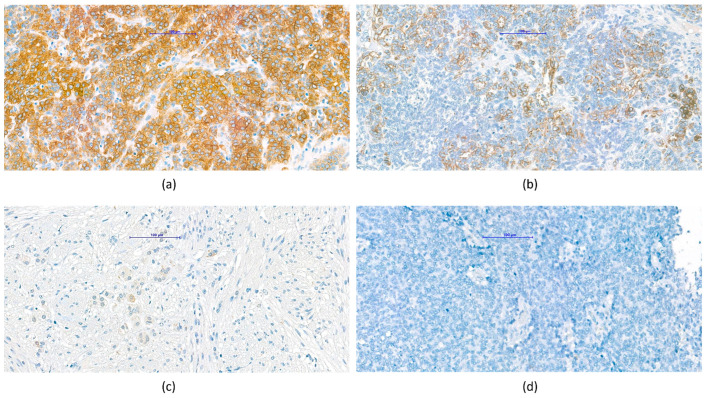
Representative vitamin D receptor (VDR) immunohistochemistry samples according to expression levels. Tumor cells showed membrane/cytoplasmic VDR expression, no nuclear positivity was observed; (**a**) score 3, high expression (VDR > 50%), (**b**) score 2, intermediate expression (10% < VDR < 50%), (**c**) score 1, low expression (VDR < 10%), (**d**) score 0, considered negative.

We determined the percentages of the appearance of VDR-expressing tumor cells on TMA blocks and scored them as mentioned above. Tumor samples with high (>50%) expression (Figure 1a) and intermediate (10–50%) expression (Figure 1b) were considered as significant VDR expression, while low (<10%) expression (Figure 1c) and non-expressing, so-called negative cells (Figure 1d) were considered as non-significant VDR expression during the later statistical analysis.

### 3.2. VDR Expression and Survival

Our first and main goal was to investigate whether lower (non-significant) VDR expression is associated with a worse (unfavorable) prognosis.

Disease outcomes were divided into favorable (complete remission, partial remission, stable disease) and unfavorable (progressive disease, exitus letalis) prognostic groups for subsequent statistical analysis.

We found that non-significant VDR expression was associated with a significantly less favorable prognosis (*p* = 0.0061) in the examined childhood solid tumors. We found that there is a clinically significant association; non-significant VDR expression has more than 14-fold odds of an unfavorable prognosis (OR = 14.74) (Table 1)

### 3.3. VDR Expression in Different Types of Childhood Solid Tumors

**Figure 2:**
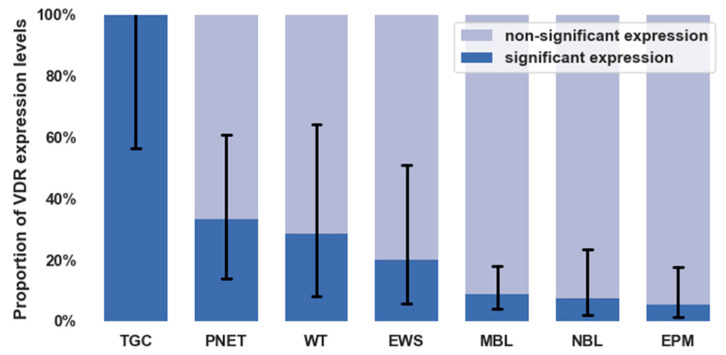
Proportion of vitamin D receptor expression levels for different diagnoses with 95% confidence intervals (VDR: vitamin D receptor; TGC: thyroid gland carcinoma; WT: Wilms’ tumor; PNET: primitive neuro-ectodermal tumors; EWS: Ewing sarcoma; NBL: neuroblastoma; MBL: medulloblastoma; EPM: ependymoma).

We aimed to evaluate whether there was a difference between the solid tumor entities examined based on the VDR expression. We found that the rate of VDR expression differed significantly between various tumor types (*p* < 0.0001). The figure shows the proportion of significant and non-significant expression with 95% confidence intervals according to tumor types (Figure 2). Numerically, the attached table shows VDR expression ratios for each tumor type (Table 2).

### 3.4. Pathological Observations Regarding VDR Characteristics of Tumor Types

#### 3.4.1. Nephroblastoma (Wilms’ Tumor)

**Figure 3:**
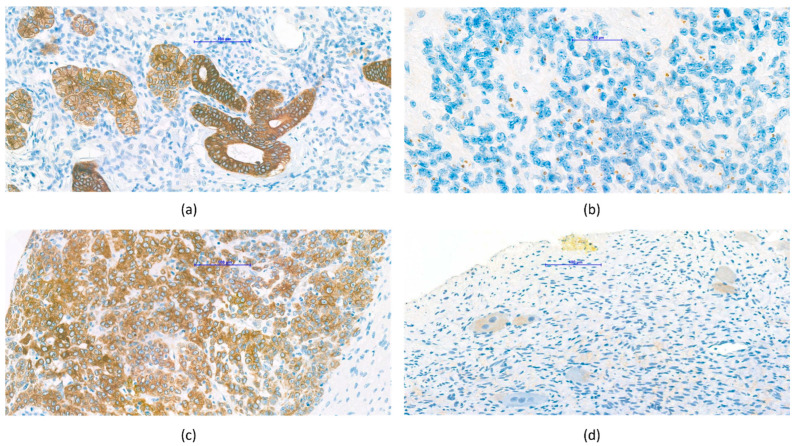
Representative VDR protein expression patterns in specific tumor subtypes. (**a**) Wilms’ tumor samples showed strong membrane/cytoplasmic expression, seen predominantly in the epithelial component. (**b**) Few ependymoma samples showed paranuclear dot-like positivity. (**c**) Hepatoblastoma samples showed strong, diffuse membrane/cytoplasmic VDR expression. (**d**) Ganglioneuroma samples showed positive ganglion cells.

Wilms’ tumor (nephroblastoma) was generally characterized by significant VDR expression, which was seen mainly on the epithelial cells of the tubules (Figure 3a).

#### 3.4.2. Central Nervous System Tumors

Based on the observations made during the evaluation, non-significant VDR expression was characteristic for ependymoma and medulloblastoma. Interestingly, paranuclear dot-like positivity was observed in some ependymoma samples (Figure 3b). In contrast, significant VDR expression was observed in PNET. Given that the current WHO tumor classification has already broken down the previous PNET category into several different entities [20], a detailed examination of these will be considered later.

#### 3.4.3. Hepatoblastoma

Relatively few (*n* = 2) hepatoblastoma samples were available (Figure 3c). Due to statistical correctness, the statistical analysis does not include the results of hepatoblastoma VDR expression measurement. However, immunohistochemistry of a total of four TMA samples from two tumor blocks revealed significant, diffuse VDR expression of the tumor cells.

#### 3.4.4. Neuroblastoma, Ganglioneuroma

In the case of neuroblastoma, it was observed that mature ganglion cells in the tumor showed mainly significant VDR expression (Figure 3d).

## 4. Discussions

An estimated 400,000 children and adolescents aged 0 to 19 years develop cancer each year. Every three minutes, a family around the world receives the heartbreaking news that their child has been diagnosed with cancer [21,22]. Cancer is a leading cause of non-accidental death for children and adolescents. The likelihood of surviving a pediatric cancer varies by country; in high-income countries, more than 80% of children with cancer are cured, whereas in many low- and middle-income countries, less than 30% of children with cancer are successfully treated [5,23].

### 4.1. Need for New Biomarkers

Childhood cancer cannot generally be prevented or identified through screening. The disease is often diagnosed at advanced stages, and this obviously affects patient outcomes negatively. There is an urgent need for identifying prognostic and therapeutic biomarkers. A prognostic biomarker is a clinical or biological characteristic that provides information on the likely course of the disease; it gives information about the outcome of the patient. A therapeutic biomarker is generally a protein that could be used as target for a therapy.

### 4.2. Role of Vitamin D in Cancer Prevention and Therapy

Vitamin D deficiency is already a widely recognized health issue, and children with cancer may be at an even higher risk than healthy children. The vitamin D receptor (VDR) is linked to cancer by an increasing body of evidence. According to the literature, people with higher serum levels of vitamin D are at lower risk for developing cancer. Vitamin D may contribute to preventing cancer by controlling cell growth, reducing inflammation, strengthening the immune system and aiding DNA repair [24,25,26,27]. According to a comprehensive analysis published in 2015, there may be a high prevalence of vitamin D deficiency and insufficiency in pediatric cancer patients, which could be linked to older age and darker skin [28]. Our previous work (2020) has shown a correlation between lower vitamin D levels in children with cancer and adverse prognosis. It suggests that supplying vitamin D is reasonable [29].

The vitamin D receptor (VDR) is a nuclear receptor superfamily ligand-dependent transcription factor [30]. The VDR is translocated into the nucleus and attaches to the vitamin D response element, triggering the transcription of specific genes when it connects to its ligand, calcitriol (1,25(OH)2D3). Activated VDR regulates several of the genes involved in a wide range of cellular functions and processes, and low vitamin D levels have been associated with cancer in humans [31,32].

### 4.3. VDR Expression and Survival

In this study, lower VDR expression was associated with a worse prognosis in various childhood tumors (significant correlation, *p* = 0.0061). The fact that an inverse association between VDR expression and tumor aggressiveness has been found suggests that VDR may be a target subjected to downregulation or ablation along the cancer progression cascade into more aggressive stages. VDR expression and activity degradation may be a common molecular change observed in a variety of tumor types, including breast, prostate and colon cancer [33,34,35].

### 4.4. Pathological Observations Regarding VDR Characteristics of Tumor Types

A tissue-specific manner of VDR expression has been shown in childhood cancer patients. VDR expression varied depending on the type of tissue examined. This suggests that VDR may play a role in regulating cell proliferation and differentiation in various tissues.

#### 4.4.1. Nephroblastoma (Wilms’ Tumor)

A similar pattern of VDR expression to Wilms’ tumor has been described in healthy kidneys. In both nephroblastoma and intact kidneys, VDR expression is characteristic of tubular epithelial cells [36] (Figure 3a). This phenomenon raises the possibility that the presence of VDR is already characteristic in the early stage of differentiation so that nephroblasts also carry this property. It is also suggested that the favorable prognosis for nephroblastoma may be related to the VDR pattern characteristic of mature renal tissue and the signaling pathway that facilitates maturation.

#### 4.4.2. Central Nervous System Tumors

In the case of ependymoma and medulloblastoma, no significant VDR expression was detected in our studies. Interestingly, paranuclear dot-like positivity was observed in some ependymoma samples. However, a phenomenon similar to that in the localization of VDR has not been described in the literature. Our observation of negativity of VDR expression does not correlate with the previous results in the literature. On the other hand, the results of immunohistochemical investigations of PNET correlate with data from the known literature. In one study, a VDR-mediated signaling pathway involved in central nervous system differentiation was described [37]. An above-mentioned review also found that VDR plays a role in the development of the central nervous system [38]. Another study raises the use of vitamin D analogs as part of a more effective treatment for central nervous system tumors in the future [39]. The discrepancy between our results and the literature may be resolved by the fact that these malignancies have the worst prognosis, so the lack of this effect may be partly responsible for the more unfavorable outcome.

#### 4.4.3. Hepatoblastoma

The diffuse, significant VDR expression we saw in hepatoblastoma samples (Figure 3c) is correlated in some studies, where significant VDR expression was detected in healthy liver tissue [40,41] and in hepatocellular carcinoma [40]. In one study, only certain cell types (sinusoidal endothelial, Kupffer and stellate cells) within the liver were found to have significant VDR expression, while other cell types were not [42]. However, some studies consider VDR undetectable in healthy liver tissue [36]. Based on these findings, it is presumed that immunohistochemical examination of the tumor VDR pattern in hepatoblastoma is recommended, and in cases of significant VDR expression, the administration of vitamin D or its analogs may be reasonable as a part of oncotherapy.

#### 4.4.4. Neuroblastoma, Ganglioneuroma

Our observation of the tumor samples of the sympathetic nervous system where VDR expression was observed in mature components, such as ganglion cells, is consistent with previous data in the literature. An animal experiment raises the effect of vitamin D via VDR on the development of the autonomic nervous system, so it may be possible that, like the healthy peripheral sympathetic nervous system, cells within the tumor mature that have VDR [43]. In a study of neuronal maturation, differentiation in the cell population was achieved by VDR overexpression on NBL cell lines [38]. A respected study of the human neuroblastoma cell line identified the exact signaling pathway through which VDR exerts its antiproliferative effect. On the basis of the results of the present study, the authors propose that vitamin D and its analogs put forward a new possible target for an anticancer therapy of human neuroblastoma [44].

## 5. Conclusions

In conclusion, VDR expression measured by IHC staining is inversely associated with aggressive characteristics in different childhood cancers. The downregulation of VDR expression in more aggressive childhood cancers suggests that functional vitamin D activity may slow or block cancer progression. However, this should be taken with a caution of reverse causality.

According to the findings, increased VDR expression in childhood solid tumor cells is linked to a favorable prognosis, i.e., a lower chance of cancer death. This provides the option of identifying high-risk patients who could benefit from the use of the VDR profile of the tumor as a biomarker and therapeutic alternative (Vitamin D, analogs). It could be an additional therapeutic option given in combination with the first-line oncology treatment.

## Figures and Tables

**Table 1:** The table below shows the number of samples belonging to each VDR expression and the prognostic group. A significant association (*p* = 0.0061) is described between lower VDR expression in tumor samples and poor outcomes. For the statistical analysis, tumor samples and the survival datasets of 110 children were available.

	Favorable Prognosis	Unfavorable Prognosis
Significant VDR expression	18	0
Non-significant VDR expression	66	26

**Table 2:** The table below lists the examined tumor types. Case numbers in each category are shown in the second column. Significant and non-significant VDR expression rates with 95% confidence intervals are shown in the third column by tumor type.

Tumor Types	Significant VDR Expression	Non-Significant VDR Expression
Neuroblastoma	2	7.4% (2.1–23.4)
Ewing sarcoma	2	20.0% (5.7–51.0)
Wilms’ tumor	2	28.6% (8.2–64.1)
Thyroid carcinoma	5	100.0% (56.6–100)
Ependymoma	2	5.4% (1.5–17.7)
Medulloblastoma	6	8.8% (4.1–17.9)
PNET	4	33.3% (13.8–60.9)

## Data Availability

The data presented in this study are available on request from the corresponding author.

## Supplementary Materials

The following supporting information can be downloaded at: https://www.mdpi.com/article/10.3390/cancers14143295/s1, Table S1: Pathological examination and clinical data.

## Abbreviations

VDR	vitamin D receptor
WHO	World Health Organization
PNET	primitive neuro-ectodermal tumor
TMA	tissue microarray
OR	odds ratio
DNA	deoxyribonucleic acid
IHC	immunohistochemistry
NBL	neuroblastoma
TGC	thyroid gland carcinoma
WT	Wilms’ tumor
EWS	Ewing sarcoma
MBL	medulloblastoma
EPM	ependymoma
